# Virulent Synergistic Effect between *Enterococcus faecalis* and *Escherichia coli* Assayed by Using the *Caenorhabditis elegans* Model

**DOI:** 10.1371/journal.pone.0003370

**Published:** 2008-10-09

**Authors:** Jean-Philippe Lavigne, Marie-Hélène Nicolas-Chanoine, Gisèle Bourg, Jérôme Moreau, Albert Sotto

**Affiliations:** 1 Institut National de la Santé et de la Recherche Médicale, ESPRI 26, Université Montpellier 1, UFR de Médecine, Nîmes, France; 2 Laboratoire de Bactériologie, Virologie, Parasitologie, CHU Caremeau, Nîmes, France; 3 Service de Microbiologie, Hôpital AP-HP Beaujon, Clichy, France; 4 Institut National de la Santé et de la Recherche Médicale, U773, Université Paris 7, Faculté de Médecine D. Diderot, Paris, France; 5 Equipe Ecologie-Evolution UMR 5561 Biogéosciences, Université de Bourgogne, Dijon, France; Massachusetts General Hospital, United States of America

## Abstract

**Background:**

The role of enterococci in the pathogenesis of polymicrobial infections is still debated. The purpose of this study was to evaluate the effect of virulent enterococci in the presence or absence of *Escherichia coli* strains in the in vivo *Caenorhabditis elegans* model.

**Principal Findings:**

This study demonstrated that there was a synergistic effect on virulence when an association of enterococci and *E. coli* (LT50 = 1.6 days±0.1 according to the tested strains and death of nematodes in 4 days±0.5) was tested in comparison with enterococci alone (LT50 = 4.6 days±0.1 and death in 10.4 days±0.6) or *E. coli* alone (LT50 = 2.1±0.9 and deaths 6.6±0.6) (p<0.001). In addition, there was a relation between the virulence of *E. faecalis* strains alone and the virulence potential of the association with *E. coli* strains. Finally, in the presence of avirulent *E. coli* strains, enterococci have no effect (LT50 = 4.3±0.5 and deaths in 10.8±0.8), independently of the level of their own virulence, demonstrating that the ‘enterococci effect’ only occurred in the presence of virulent *E. coli* strains.

**Conclusion:**

This study allows a better understanding of a bacterial cooperation. Moreover, it could help to optimize the antibiotic regimen during polymicrobial infections.

## Introduction

The role played by enterococci in the pathogenesis of intra-abdominal infections is still debated because enterococci are frequently isolated from the polymicrobial flora [1]–[3]. In order to get insight into the role of enterococci in polymicrobial infections, Carbon et al. developed animal models and showed that enterococci might have a synergistic effect on the pathogenic traits of associated microorganisms, particularly *Escherichia coli* [4]–[6]. In the present study, we used another in vivo model, namely the *Caenorhabditis elegans* model, which was previously used to explore virulence of various micro-organisms (*Serratia marcescens*, *Salmonella enterica*, *Pseudomonas aeruginosa*, *Burkholderia cepacia*, *E. coli, Staphylococcus aureus* and *Streptococcus pyogenes* [7]–[13]). Garsin et al. also used this model to investigate *Enterococcus faecalis* related virulence factors [14], [15]. To our knowledge, the present study is the first one in which the *C. elegans* model was used to assess the virulence of associated microorganisms such as *E. coli* and *E. faecalis*.

## Results

The virulence factor-encoding genes detected in the different strains of *E. faecalis* are listed in Table 1.

**Table 1:** Detection of virulence factor (VF)-encoding genes (*asa1, gelE, cylA, esp* and *hyl*) and production of hemolysin (*hly*B), gelatinase and aggregation substance among *E. faecalis* strains.

Strain	Origin	*gelE*	*cylA*	*esp*	*hyl*	*asa1*	VF score	Clumping factor	*hly*B
NEF26	UTI	+ (+)	−	+	−	+	3	+	(−)
NEF42	UTI	− (−)	+	−	−	−	1	NA	(−)
NEF889	UTI	+ (−)	+	+	−	+	4	+	(−)
NEF21895	UTI	+ (+)	−	+	−	−	2	NA	(−)
NEF17649	UTI	− (−)	−	+	−	−	1	NA	(−)
NEF64	Bacteraemia	+ (+)	−	−	+	+	3	+	(+)
NEF48015	Bacteraemia	+ (+)	−	−	−	−	1	NA	(−)
NEF27828	UTI	− (−)	−	−	−	+	1	−	(−)

Symbols in parentheses indicate expression of the gene: +, presence of virulence factor; −, absence of virulence factor

NA, not applicable

**Figure 1:**
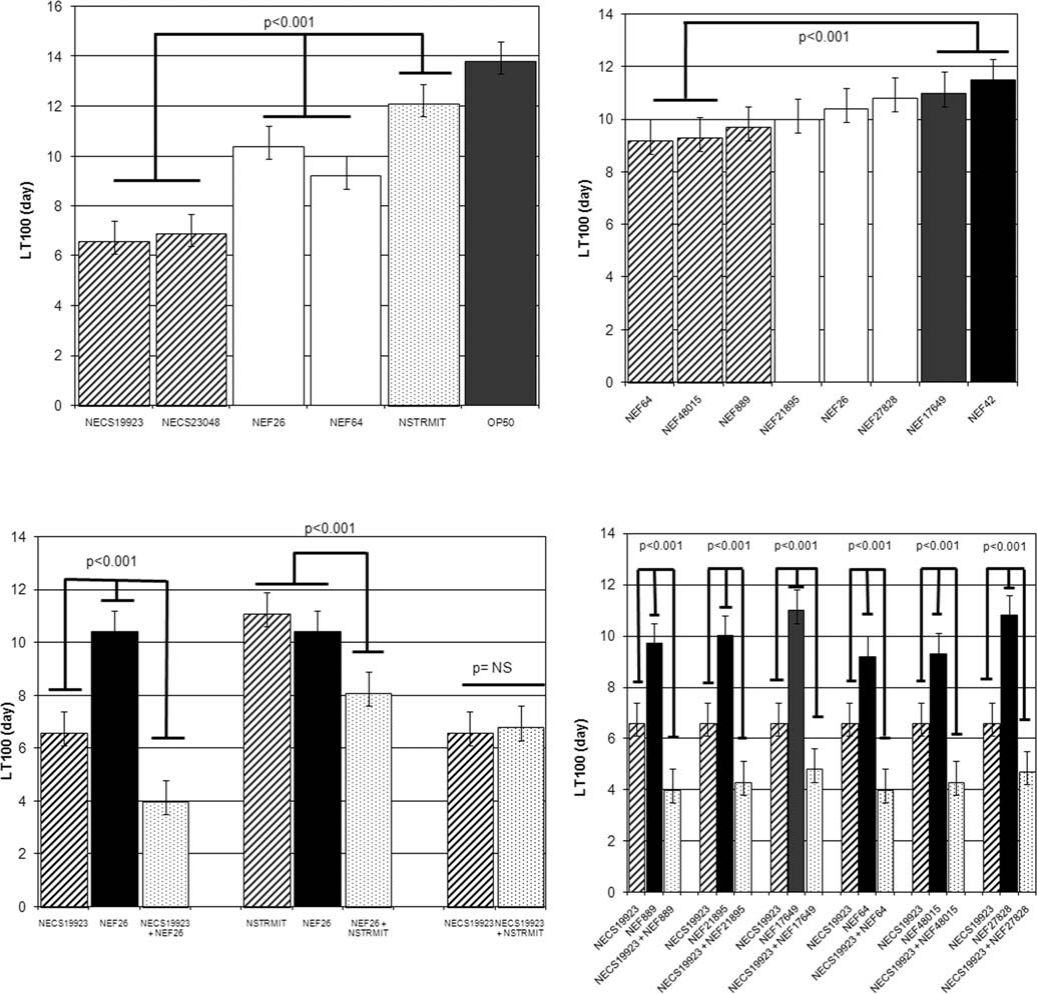
In vivo kinetics of killing of *C. elegans* infected by *E. coli, E. faecalis* and *S. mitis* strains. A. Comparison of LT100 between the different strains tested alone. B. Comparison of LT100 between *E. faecalis* strains tested alone. C. Comparison of LT100 with strains tested alone or in association. NEF26 was representative of the different *E. faecalis* strains. D. Comparison of LT100 between *E. coli* NECS19923 strain and the different *E. faecalis* strains tested in association. The results are representative of at least four independent trials for each group of strains. NS: non significant.

All *E. faecalis, E. coli* and *S. mitis* strains tested alone killed *C. elegans* (Fig. 1A). However, the two uropathogenic *E. coli* strains were significantly more virulent (LT50 of 2 days) than both *E. faecalis* strains and the *S. mitis* strain (LT50 varying from 4 to 5 days) (p<0.001). Interestingly, the bacteraemic *E. faecalis* strains (NEF64, NEF48015) were significantly more virulent (LT50 4.1 days±0.1) than UTI strains (LT50 4.4 to 4.8 days±0.1), even though they harboured no more VFs than UTI strains (p<0.001) (Fig. 1B).

When *E. coli* and *E. faecalis* strains were associated, a virulent synergistic effect was observed, irrespective at the specific uropathogenic *E. coli* and *E. feacalis* strains associated (Fig. 1C and 1D). The LT50s obtained with the strain associations varied between 1.5 and 1.7 days and were significantly shorter than the LT50s observed with *E. coli* strains alone (p<0.001). To demonstrate the role of enterocci in the increase in virulence, the *Enterococcus* spp. strain was replaced by the *S. mitis* strain (which harboured *ply* gene). In the nematode killing model, *E. faecalis* and *S. mitis* strains presented a similar virulence (LT50_NSTRMIT_ 5.4 days vs LT50_NEF26_ 4.6 days; (p: not significant)) (Fig. 1A). The association of *E. coli* NECS19923 and the *S. mitis* strain did not increase the virulence observed in the nematode model (LT50_NECS19923+NSTRMIT_ 2.2 days) in comparison with the virulence obtained with *E. coli* alone (LT50_NECS19923_ 2.1 days) (Fig. 1C). Conversely, the virulence of the association of the *S. mitis* strain with *E. faecalis* (LT50_NEF26+NSTRMIT_ 3.6 days) was significantly higher than the virulence observed with either *S. mitis* alone (LT50_NSTRMIT_ 5.4 days) and enterocci alone (LT50_NEF26_ 4.6 days) (p<0.001) (Fig. 1C). All these findings strongly suggested the essential role of *E. faecalis* strains in the synergistic effect. However this synergistic effect of the association between *E. coli* and *E. faecalis* was only detected in presence of virulent *E. coli* strains. This was suggested by the LT50s observed when *E. faecalis* strains were associated with the avirulent *E. coli* OP50 strain (LT50_NEF26_ 4.5 days vs LT50_NEF26+OP50_ 4.3 and LT100_NEF26_ 10.4 days vs LT100_NEF26+OP50_ 10.8; p: not significant).

**Figure 2:**
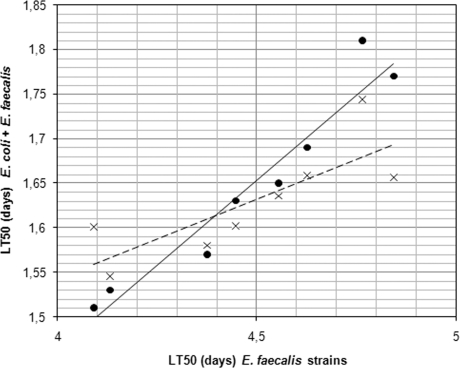
Correlation between virulence of *E. faecalis* strains alone (expressed by LT50s in days) and in combination with *E. coli* strains. The linear regression model confirmed a linear relation. Dot corresponds to *E. faecalis* strains associated with NECS23048; cross corresponds to *E. faecalis* strains associated with NECS19923. The line and the dotted line established the linear regression of NECS23048 and NECS19923, respectively.

Moreover, as indicated in Fig. 2, a correlation was observed between the value of the LT50 of the *E. faecalis strains* tested alone and the value of the LT50 of the association of *E. coli* and *E. faecalis* strains.

To verify the ingestion of *E. coli, S. mitis* and *E. faecalis* strains and the proliferation in the *C. elegans* intestine, L4 *C. elegans* were fed on a mixed lawn of *E. faecalis* and *E. coli, E. faecalis* and *S. mitis* or *S. mitis* and *E. coli* at a same ratio 1:1. At 72 h, the nematodes were washed to remove bacteria from their surfaces and then disrupted to recover bacteria from inside their digestive. The number of *E. faecalis, S. mitis* and *E. coli* CFU within the nematode gut varied between 10^5^ and 10^6^ bacteria per worm between each combination without statistical difference (Table 2).

**Table 2:** Evaluation of the number of bacteria within the *C. elegans* digestive tract.

Strains	Median CFU's of strains/ nematode after 72 h	
	Strain 1*	Strain 2
*E. coli* NECS19923^ψ^	1.4 10^6^ [0.9–1.7 10^6^]	–
*E. faecalis* NEF26^ψ^	6 10^5^ [5.5–6.7 10^5^]	–
*E. faecalis* NEF42^ψ^	4.5 10^5^ [4.0–5.1 10^5^]	–
*S. mitis* NSTRMIT^ψ^	2.2 10^5^ [1.9–3.0 10^5^]	–
NEF26^ψ^ + NECS19923	5 10^5^ [4.5–5.5 10^5^]	8 10^5^ [7.5 10^5^–10^6^]
NEF42^ψ^ + NECS19923	3.9 10^5^ [3.0–4.6 10^5^]	1.1 10^6^ [0.8–1.3 10^6^]
NEF26^ψ^ + NSTRMIT	5.1 10^5^ [4.4–6.0 10^5^]	2.7 10^5^ [1.9–3 10^5^]
NEF42^ψ^ + NSTRMIT	4.3 10^5^ [3.6–5.1 10^5^]	3.2 10^5^ [2.4–4.1 10^5^]
NECS19923^ψ^ + NSTRMIT	10^6^ [0.8–1.3 10^6^]	1.9 10^5^ [0.9–2.7 10^5^]
*E. faecium,* control strain ^ψ^	10^1^ [0.5–1.3 10^1^]	–

After 72 hr of infection, the *C. elegans* were washed and ground, and dilutions of the resulting suspension were plated on selective media. The number of CFU per worm of the different strains alone or in combination was calculated. Three replicates were performed for each bacterial combination. *E. faecium* was used as negative control. No statistical difference could be demonstrated.

Range of values are indicated into [square brackets]

* Strain 1 corresponded to the first strain with ^ψ^; strain 2 corresponded to the second strain when associations were tested.

## Discussion

Both the clinical significance of enterococci in peritonitis and the use of empiric anti-enterococcal coverage have been the subject of a long-lasting and ongoing debate [16]–[19]. In a literature review recently published by Harbarth and Uckay, was presented evidence arguing in favour of using empirical therapy with enterococcal coverage for intra-abdominal infections among certain patients considered as high-risk [19].

The pathogenic role of enterococci has been studied in animal models. Onderdonk et al. showed in a model of peritonitis in rat that enterococci inoculated alone induced neither early death nor later abscesses [20]. They demonstrated a synergic effect between enterococci and anaerobes but not between enterocci and *E. coli*. Later, Carbon et al. underlined several important points by using another model of experimental intra-abdominal infection. They studied the pathophysiological role of enterococci in a non fatal model of peritonitis in rats by implanting a gelatin capsule containing *E. coli* and *Bacteroides fragilis* with or without increasing concentrations of *E. faecalis* or heat-inactivated enterococci [6]. Effects were measured after several hours and several days following inoculation. Thus, they observed that the highest concentration of enterococci was correlated with an increase in *E. coli* and proinflammatory cytokines (tumor necrosis factor and interleukin) concentrations [21]. They suggested that enterococci might limit phagocytosis and intracellular death of other pathogens, particularly *E. coli*. In another study, the same authors evaluated the effects of *E. faecalis* strains containing various combinations of virulent factors in mouse and rat models of peritonitis. However, no conclusion was provided because of variable findings [4].

Currently, ten of enterococcal virulence factors are known. Their exact role in pathogenic mechanisms remains dubious and in several publications no conclusions were possible or were contradictory. In this study we screened for the six most studied virulence factors. Aggregation substance, encoded by *asa1*, is a pheromone-inducible protein that increases (i) bacterial adherence to renal tubular cells and heart endocardial cells, (ii) internalization by intestinal cells, and (iii) the valvular vegetation mass in an animal model of endocarditis. Gelatinase, encoded by *gelE*, is an extracellular zinc endopeptidase that hydrolyzes collagen, gelatin, and small peptides and that has been shown to exacerbate endocarditis in an animal model [22]. The production of cytolysin (encoded by *cylA*) has also been shown to significantly worsen the severity of endocarditis and endophthalmitis in animal models and is a virulence factor that affects *C. elegans* killing [14], [22]. The enterococcal surface protein, encoded by *esp*, is associated with increased virulence, colonization and persistence in the urinary tract, and biofilm formation. Hyaluronidase, encoded by *hyl*, contributes to invasion of the nasopharynx and pneumococcal pneumonia [22]. Hemolysin, encoded by *hlyB,* is a cytotoxic protein that contributes to the destruction of red blood cells. Vergis et al. showed that the presence of gelatinase, hemolysin and enterococcal surface protein singly or in combination was not associated with mortality among patients with bacteremia due to *E. faecalis* [23]. In this work, the most virulent *E. faecalis* strains did not necessarily contain the most number of virulence genes and no virulent ‘markers’ could be identified, including Cyl, that caused faster killing in the nematode model [14]. This confirms the difficulty in understanding the virulence of enterococci.

In the present study, using for the first time the *C. elegans* model to investigate microorganism association, we clearly demonstrated a synergistic interaction between *E. coli* and *E. faecalis*, two bacteria frequently isolated from peritonitis pus and UTI and also known to independently induce an intestinal infection in this nematode model [9], [14]. The LT50s of the associations of *E. faecalis* and *E. coli* strains were definitively smaller than the LT50s observed with each microorganism tested alone. Interestingly the higher the level of virulence of the *E. faecalis* strain, the more virulence was observed in the bacterial association with *E. coli*. However, the synergic effect did not seem to be influenced by the number of virulence factors of *E. faecalis* as described previously [24], but seemed to be influenced by the virulence of *E. coli*. Indeed no synergistic effect was observed between *E. faecalis* and *E. coli* OP50, a strain with low virulence potential. Such findings are in accordance with a recent data that have shown an association between the presence of numerous virulence factors, specific O types, B2 phylogenetic group and virulence in the *C. elegans* and murine models among uropathogenic *E. coli* strains [25], [26]. Finally the determination of the number of bacteria within the *C. elegans* digestive tract validated the presence and the overgrowth of *E. faecalis*, *E. coli* and *S. mitis* in the intestine of nematodes. This result demonstrates the establishment of a true infection validating our results. As previously demonstrated, *E. faecalis* not only accumulates in the intestinal lumen but proliferates in the gut, resulting in a long-lasting infection that persists for the entire lifespan of the worms [14].

Many questions concerning the exact pathogenicity of enterococci remain unanswered. However, this model suggests the role of enterococci in increasing virulence in a polymicrobial infection. This is consistent with Carbon's model. This study allows us to better understand bacterial cooperation and it could help to optimize the antibiotic regimen during polymicrobial infections.

## Materials and Methods

### Bacterial strains and growth conditions

Eight *E. faecalis* strains (NEF26, NEF42, NEF889, NEF21895, NEF17649, NEF64, NEF48015, NEF27828) isolated from the blood and urinary tract infections [27] (Table 1) were studied alone and combined with other microorganisms. These microorganisms, which also were studied alone, comprised *Streptococcus mitis* strain NSTRMIT isolated from the blood and *E. coli* strains NECS19923, NECS23048 both isolated from UTIs [9]. Two others strains were used as controls in the different experiments namely *E. faecalis* JH2-2 (control strain for expression of aggregation substance) and the avirulent *E. coli* OP50 (an international standard food for nematodes without known virulence factors). Bacteria were grown in trypticase soy broth or agar (TSA) or Columbia agar gelose with 5% fresh sheep blood (bioMerieux, Marnes La Coquette, France) at 37°C in 5% CO_2_.

### Virulence genotyping

*E. coli* strains used in this study had been previously characterized for the virulence genotype [9]. NECS19923 and NECS23048 belonged to B2 group and presented virulence profiles: *pap*GII, *pap*A, *pap*C, *sfaS*, *focG*, *afa/draBC*, *fimH*, *hlyA*, *cnf1*, *iutA*, *kpsMT*K1, *kpsMT*II, *traT, iroN*, *malX, irp2* and *pap*GIII, *pap*A, *pap*C, *focG*, *afa/draBC*, *fimH*, *hlyA*, *cnf1*, *iutA*, *kpsMT*K1, *kpsMT*II, *traT, iroN*, *malX, irp2*, respectively. *E. faecalis* and *S. mitis* isolates were tested by PCR for the presence of a panel of genes encoding known virulence factors (VFs). For *E. faecalis*, the multiplex PCR used the panel of 5 genes (*asa1, gelE, cylA, esp* and *hyl*) previously described [22]. For *S. mitis*, we used *ply* PCR to determine the presence of pneumolysin gene [28]. Production of gelatinase was determined by using TSA supplemented with 1.5% skimmed milk. The presence of a clear halo around colonies after overnight incubation at 37°C was considered to be a positive result [29]. Haemolysin production was evaluated on Columbia agar gelose with 5% fresh sheep blood. Zones of clearing around colonies after 18 h at 37°C indicated production of β-hemolysin [24]. Expression of aggregation substance was determined for PCR-positive strains by the clumping assay as previously described [21], [30]. *E. faecalis* JH2-2 was used as control.

### Nematode Killing Assay

The *C. elegans* infection assay was carried out as described by Kurz et al. [25] except that the Fer-15 mutant line, which has a temperature sensitive fertility defect, were used rather than wild type N2 worms. Fer-15 was provided by the *Caenorhabditis* Genetics Center, which is funded by the NIH National Center for Research Resources (NCRR). To synchronize the growth of worms, eggs were collected using the hypochlorite method. Overnight cultures in brain heart infusion (BHI) medium of *E. coli, S. mitis and E. faecalis* strains were harvested by centrifugation, washed once and suspended in phosphate buffered saline solution (PBS) at pH 7.0 at a concentration of 10^5^ CFU/ml. BHI agar plates were inoculated with 10 μl of each strain and incubated at 37°C for 8−10 h. Plates were allowed to cool to room temperature and seeded with L4 stage worms (20–30 per plate). Plates were then incubated at 25°C and scored each day for live worms under a stereomicroscope (Leica MS5) [9]. At least three replicates repeated 5 times were performed for each selected clone. A worm was considered dead when it no longer responded to touch. Worms that died as a result of becoming stuck to the wall of the plate were excluded from the analysis. Lethal Time 50% (LT50) and death corresponded to time (in hours) required to kill 50% and 100% of the nematode population, respectively.

### Measuring the number of bacteria within the *C. elegans* digestive tract

This assay was carried out as described by Garsin et al. [14]. Five *C. elegans* were picked at 72 h, and the surface bacteria were removed by washing the worms twice in 4 μl drops of M9 medium on a BHI agar plate containing 25 μg/ml gentamicin. The nematodes were placed in a 1.5 ml Eppendorf tube containing 20 μl of M9 medium with 1% Triton X-100 and were mechanically disrupted by using a pestle. The volume was adjusted to 50 μl with M9 medium containing 1% Triton X-100 which was diluted and plated on BHI agar containing 50 μg/ml ampicillin. At least three replicates were performed for each bacterial combination.

### Statistical analysis

To compare the entire survival curves in nematode killing assays, we used a Cox regression. In order to perform pairwise comparison between two different strains, we used a log rank test. The analysis was carried out using SPSS 6.1.1 (SPSS Inc., Chicago, IL, USA).
